# Design of the Tocilizumab in Giant Cell Arteritis Trial

**DOI:** 10.1155/2013/912562

**Published:** 2013-04-07

**Authors:** Sebastian H. Unizony, Bhaskar Dasgupta, Elena Fisheleva, Lucy Rowell, Georg Schett, Robert Spiera, Jochen Zwerina, Olivier Harari, John H. Stone

**Affiliations:** ^1^Massachusetts General Hospital, Harvard Medical School, Boston, MA 02114, USA; ^2^Southend Hospital, Westcliff-on-Sea, Essex, SS0 0RY, UK; ^3^Hoffmann-LaRoche Pharmaceuticals Inc., Shire Park, Welwyn Garden City AL7 1TW, UK; ^4^University of Erlangen-Nuremberg, Krankenhausstrasse 12, 91054 Erlangen, Germany; ^5^Hospital for Special Surgery, Cornell, New York, NY 10021, USA

## Abstract

*Overview*. The GiACTA trial is a multicenter, randomized, double-blind, and placebo-controlled study designed to test the ability of tocilizumab (TCZ), an interleukin (IL)-6 receptor antagonist, to maintain disease remission in patients with giant cell arteritis (GCA). *Design*. Approximately 100 centers will enroll 250 patients with active disease. The trial consists of a 52-week blinded treatment phase followed by 104 weeks of open-label extension. Patients will be randomized into one of four groups. Group A (TCZ 162 mg weekly plus a 6-month prednisone-taper); group B (TCZ 162 mg every other week plus a 6-month prednisone-taper); group C (placebo plus a 6-month prednisone-taper); and group D (placebo plus a 12-month prednisone taper). We hypothesize that patients assigned to TCZ in addition to a 6-month prednisone course are more likely to achieve the primary efficacy endpoint of sustained remission (SR) at 52 weeks compared with those assigned to a 6-month prednisone course alone, thus potentially minimizing the long-term adverse effects of corticosteroids. *Conclusion*. GiACTA will test the hypothesis that interference with IL-6 signaling exerts a beneficial effect on patients with GCA. The objective of this paper is to describe the design of the trial and address major issues related to its development.

## 1. Introduction

Giant cell arteritis (GCA, temporal arteritis) is an inflammatory disease of medium- and large-sized arteries that affects individuals older than 50 years of age [1]. The disease commonly involves the aorta, great vessels, and the extracranial branches of the carotid arteries. GCA is the most common primary form of vasculitis in Western countries and has a prevalence that ranges from 24 to 280 cases per 100,000 in individuals older than 50 years [2–4]. Its clinical presentation consists of constitutional symptoms, headaches, ischemia-related visual manifestations, jaw claudication, and polymyalgia rheumatica. The inflammatory markers (erythrocyte sedimentation rate [ESR] and C-reactive protein [CRP]) are elevated in the vast majority of the cases during active disease [5].

GCA and its current treatment strategies carry a substantial morbidity burden. The most feared consequence of the disease is blindness [6], but multiple complications can ensue (e.g., tongue necrosis, aortic aneurysm, stroke, or myocardial infarction). Corticosteroids (CS), the mainstay of treatment, control systemic inflammation effectively and prevent acute damage (i.e., vision loss) but generally fail to cure GCA or induce long-term CS-free remissions. Fifty to 80% of the patients relapse upon dose reduction and therefore require long-term treatment courses that are associated with toxicity in nearly all cases (i.e., hypertension, infection, fragility fractures, cataracts, gastrointestinal bleeding, weight gain, diabetes mellitus, and psychosis) [7, 8].

Attempts to control disease activity and spare CS with other agents have generally failed [9–11]. Trials using methotrexate (MTX) have produced conflicting results [12–14], and studies of tumor necrosis factor inhibitors have been negative [15, 16]. Thus, an agent capable of maintaining disease remission after the discontinuation of high-dose CS is still lacking. Based on results from the open-label use of the IL-6R antagonist tocilizumab (TCZ, Actemra; Roche) for the treatment of GCA [17–25] and data generated in TCZ trials for rheumatoid arthritis (RA) [26–29], a phase III randomized, double-blind, and placebo-controlled trial of TCZ for GCA has been initiated. The objectives of this paper are to describe the design elements of the GiACTA trial and to discuss issues related to the development of the study.

## 2. Design and Methods

### 2.1. Rationale for Using IL-6 Blockade in GCA

The cellular branch of the adaptive and innate immune systems appears to be central to the pathogenesis of GCA, even though the antigen(s) that trigger the disease remain unknown [30, 31]. In untreated patients, an expanded repertoire of autoreactive CD_4_-positive T lymphocytes, including IFN-*γ* producing T helper (Th) 1 cells and IL-17-secreting Th17 cells, orchestrates the formation of granulomatous vascular inflammation [32–35]. In contrast, the number of Foxp3+ regulatory T cells (Treg), which normally serve to limit immune responses, is decreased [33, 35]. 

IL-6 is a pleiotropic cytokine produced by T cells, B cells, macrophages, endothelial cells, and fibroblasts upon different stimuli [36]. Under physiologic conditions, IL-6 triggers the synthesis of acute phase proteins, promotes the transition from acute to chronic inflammation, and facilitates the development of specific immunity. IL-6 participates in the activation of T cells, the terminal differentiation of B cells, the survival of plasmocytes, the differentiation of Th17 lymphocytes [37], the inhibition of Treg-cell differentiation and function [38], and the induction of a proinflammatory phenotype among monocytes/macrophages, endothelial, and stromal cells. Thus, the IL-6 pathway is located at the intersection of the innate and acquired immune systems and, if dysregulated, has the potential to perpetuate inflammatory responses.

In GCA patients, IL-6 is up regulated within inflamed arteries [39–41], and its concentration in the peripheral circulation is elevated [42, 43]. Serum IL-6 levels mirror disease activity and decline with adequate CS treatment [44, 45]. We speculate that IL-6R blockade with tocilizumab may ameliorate vascular inflammation through several mechanisms: (a) altering upstream differentiation of autoreactive lymphocytes [46–48]; (b) promoting the generation of Treg cells [49]; and (c) targeting downstream aspects of the inflammatory cascade [40].

In published reports, approximately two dozens of patients with GCA have received TCZ [17–25]. Patients responded well, and no limiting safety concerns were noted. In GCA patients, pre- and postdose CRP levels were similar to those seen in RA, and the normalization of this inflammatory parameter was sustained.

### 2.2. Study Design

**Figure 1:**
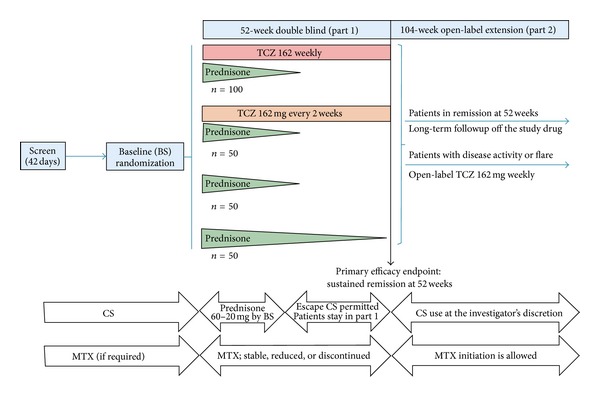
Study schema.

GiACTA is designed to evaluate the safety and efficacy of TCZ for the treatment of GCA. Two hundred and fifty (250) patients will be enrolled and assigned to one of four treatment arms. The trial will consist of a 52-week blinded period (Part 1), followed by a 104-week open-label extension (Part 2) (Figure 1).

Two subcutaneous (SC) doses of TCZ (162 mg every week [162qw] and 162 mg every other week [162q2w]) will be compared to placebo. All patients will receive background CS therapy. Three groups (A, B, and C) will follow a prespecified prednisone-taper regimen over 26 weeks, and a fourth group (group D), designed to reflect an alternate standard of care, will receive a 52-week prednisone taper (see Figure 1 and Section 2.8). 

The primary efficacy endpoint, sustained remission (SR), will be evaluated at 52 weeks. Remission is defined as the absence of signs and symptoms attributable to GCA and normalization of ESR (<30 mm/Hr) and CRP (<1 mg/dL). Other definitions used in the trial are described in Table 1.

The purpose of Part 2 is to determine the long-term safety and maintenance efficacy of TCZ, to explore a potential requirement for maintenance treatment beyond 52 weeks, and to gain insight into the long-term CS-sparing effect of IL-6R blockade. Those who achieve the primary endpoint will stop their blinded SC injections and be followed for maintenance of response. Patients with persistent disease activity or those who experience a flare after week 52 will have the option to receive open-label TCZ (162 mg weekly) with or without increase in background CS dose at the discretion of the investigator. The duration of open-label therapy will be determined by the investigator according to the patient's clinical condition. 

### 2.3. Organization and Funding

The GiACTA research group comprises 100 sites in the USA and Europe. The trial is funded by F. Hoffmann-La Roche, Ltd (Switzerland). Mechanistic studies are supported in part through a grant from the Arthritis Foundation.

### 2.4. Institutional Review Board Approval and Informed Consent

Each participating clinic will have institutional review board oversight. All participants will give written informed consent.

### 2.5. Trial Objectives and Hypothesis

The primary objective of GiACTA is to evaluate the efficacy of TCZ in combination with a six-month prednisone taper to sustain remission through 52 weeks. To meet this endpoint, a patient must maintain disease remission after remission induction by week 12 of randomization, complete the assigned prednisone-taper protocol, and not flare or require escape therapy at any time until week 52. 

The primary efficacy analysis will compare groups A and B against group C (see Section 2.7). We hypothesize that patients assigned to TCZ in addition to a 26-week prednisone course are more likely to achieve SR at 52 weeks compared with those assigned to a 26-week prednisone course alone, thus potentially minimizing the long-term adverse effects of CS.

Secondary and exploratory objectives of the trial are to evaluate safety, the impact of TCZ on cumulative CS exposure, long-term remission maintenance beyond 52 weeks, patient-reported outcomes, and to compare the TCZ arms against the 52-week prednisone taper. Pharmacokinetic-pharmacodynamic (PK-PD) studies, immunogenicity assays, and mechanistic investigations will also be performed.

### 2.6. Eligibility Criteria and Assessment of Disease Activity

The eligibility criteria for the trial are summarized in Table 2. Patients will be required to have active disease within 6 weeks prior to the baseline visit. New onset and relapsed/refractory GCA patients will be eligible. The number of relapsed/refractory subjects will be capped at 70%. Active disease for enrollment is defined as the presence of unequivocal clinical signs and symptoms attributable to GCA and increased levels of circulating inflammatory markers (ESR  ≥30 mm/hr and/or CRP  ≥1 mg/dL). All patients will receive background prednisone therapy and follow a prespecified tapering protocol (see Table 3 and Section 2.8).

### 2.7. Assignment to Study Groups

Two hundred and fifty patients will be randomized through an interactive voice response system (IVRS) in a 2 : 1 : 1 : 1 ratio to the different study arms as follows (Figure 1):

- TCZ 162qw + 26 weeks of prednisone + 26 weeks of prednisone placebo (*n* = 100);
- TCZ 162q2w + 26 weeks of prednisone + 26 weeks of prednisone placebo (*n* = 50);
- TCZ-placebo + 26 weeks of prednisone + 26 weeks of prednisone placebo (*n* = 50); and
- TCZ-placebo + 52 weeks of prednisone (*n* = 50).

To ensure balance across groups, the randomization will be stratified by baseline prednisone dose (<30 mg/day or ≥30 mg/day) and whether the patients present with new onset or refractory/relapsed disease.

Subjects will receive either TCZ or matching TCZ placebo by SC injection once a week. The dose modification guidelines and risk mitigation strategy used for TCZ in RA will be implemented in GIACTA (Actemra [tocilizumab] US Package Insert, RoActemra [tocilizumab] European Union Summary of Product). Measures designed to protect against unblinding are addressed in the Discussion section. 

### 2.8. Prednisone Regimens

#### 2.8.1. Assignment of the Initial Prednisone Dose

The investigator will determine the initial prednisone dose from the time of enrollment based on the disease history (new onset or refractory/relapsing disease), severity of activity (e.g., visual symptoms), and comorbidities (e.g., diabetes mellitus). By definition, patients with refractory/relapsing GCA will have active disease despite previous or concurrent CS therapy. In the last case, an increase in CS dose will be required upon enrollment. Permitted ranges of starting doses for any patient will be 20–60 mg daily. From the dose chosen at baseline, the patients will proceed with a protocol-defined tapering schedule depending on the group assigned (Table 3). “Pulse” intravenous doses of glucocorticoids defined as methylprednisolone >100 mg/day will not be permitted if given within 6 weeks prior to enrollment.

#### 2.8.2. Short and Long Prednisone-Taper Protocols

There will be two standardized prednisone-taper regimens: one that extends for 26 weeks (groups A, B, and C) and the other for 52 weeks (group D) (Figure 1 and Table 3). The total duration of prednisone therapy in each particular case will depend on the initial dose required by the patient as judged by the investigator at enrollment. 

#### 2.8.3. Blinding of Prednisone Treatments

The prednisone tapers will have an open-label phase and a blinded phase (Table 3). Prednisone doses between 60 mg and 20 mg will be administered in an open-label fashion. In order to prevent unblinding due to the different taper lengths, prednisone dosages below 20 mg will be provided in numbered blister packs for blinded administration. Depending on the patient's assignment to either the six- or twelve-month taper regimen, the daily encapsulated dose may contain active prednisone, prednisone placebo, or a combination of both.

### 2.9. Concomitant Immunosuppressive Medications

Concomitant treatment with stable doses of MTX will be allowed only if started more than 6 weeks prior to the study enrollment. The dose of MTX should remain stable and not be increased throughout the screening and 52 week double-blind treatment periods. During these periods, the MTX dose may be reduced or discontinued if necessary for safety reasons, or, if in the opinion of the investigator, it is no longer required to treat the patient's GCA. Other immunosuppressive medications will not be allowed during the study (Table 2). 

### 2.10. Treatment of Flares (Escape Therapy)

Patients who cannot follow the protocol-defined prednisone taper due to disease flare will continue in the study under an investigator-defined open-label escape CS regimen in combination with double-blind injections of TCZ/TCZ placebo for the full 52 weeks. Although these patients will be deemed as nonresponders for the primary outcome, their subsequent followup and CS requirement will provide important information for the analysis of secondary and exploratory endpoints. 

### 2.11. Description of Study Assessments Efficacy Assessments

Clinical GCA activity that will be evaluated at every study visit include: fever (≥38°C or 100.4°F); symptoms of PMR; headache; temporal artery or scalp tenderness; visual signs or symptoms such as acute or subacute vision loss due to arteritic anterior ischemic optic neuropathy, transient blurry vision, amaurosis fugax, or diplopia; jaw claudication; new or worsened extremity claudication; and other features judged by the investigator to be consistent with a GCA flare.

*Laboratory Assessments. *Laboratory assessments to be obtained at different timepoints will include: complete blood counts; serum chemistry profile including renal and liver function tests; fasting serum lipids; HbA1c; hepatitis B and C serologies; and ESR and CRP. In order to maintain blinding, the investigator/efficacy assessor will be masked to the results of complete blood counts, serum chemistry, ESR, and CRP.

*Serological PD-PK Measures and Immunogenicity Assays*. Assays designed to evaluate pharmacodynamic and pharmacokinetic parameters of TCZ will include concentrations of CRP, IL-6, sIL-6R, TCZ, and anti-TCZ antibodies.

*Patient-Reported Outcomes*. Planned patient-reported outcomes include the patient global assessment of disease activity (PGA) using a VAS scale and the SF-36, FACIT Fatigue, and EQ-5D questionnaires.

*Safety Assessments*. Review of adverse events, vital signs, concomitant medications, and laboratory data will be performed.

*Mechanism of Action Studies*. Serum, RNA, and DNA samples will be collected at defined timepoints for mechanistic studies.

### 2.12. Outcome Measures and Study Endpoints

The efficacy outcome measures include the assessment of disease remission based on signs and symptoms of active GCA; ESR and CRP; adherence to the prespecified prednisone taper; the number of flares and the time to disease flare postremission; the cumulative CS dose; and patient-reported quality of life (QoL) assessments.

The safety outcome measures include the incidence, nature, and severity of adverse events; laboratory abnormalities (i.e., cytopenias, liver function tests abnormalities, and lipid abnormalities), and immunogenicity.

The definition of the primary endpoint, SR at week 52, has four aspects:

1. achievement of disease remission not later than week 12 after randomization;
2. absence of disease flare through week 52;
3. completion of the assigned prednisone taper protocol;
4. absence of the need for escape therapy (prednisone increase) at any time during the 52 weeks after randomization.

Remission is defined as the absence of signs and symptoms attributable to GCA and normalization of ESR (<30 mm/Hr) and CRP (<1 mg/dL).

Patients who do not achieve remission within 12 weeks; do not reach a prednisone dose of zero by the predefined time (Table 3); require escape therapy; or are withdrawn early from the study for any reason will be deemed as nonresponders in the analysis of the primary endpoint. 

### 2.13. Analysis

#### 2.13.1. Efficacy Analysis

The analysis of the data will be performed on an intention-to-treat basis, including all randomized subjects.

For the primary efficacy endpoint, the TCZ treatment groups will be compared to placebo (in combination with 26 weeks prednisone-taper regimen) using a Cochran-Mantel-Haenszel test, adjusting for new onset versus refractory/relapsing disease and starting prednisone dose (≤30 mg/day, >30 mg/day).

For the analysis of secondary endpoints, all continuous endpoints such as SF-36, PGA, and cumulative CS dose will be analyzed using a maximum likelihood repeated measures method to compare the change from baseline to the week 52 value. The analyses will be adjusted for the stratification factors applied at randomization, as well as the baseline value for the parameter being tested. The time to disease flare will be analyzed using a Cox proportional hazards model including stratification factors. The secondary endpoints will be tested using a prespecified fixed sequence method, and adjustments required to control for multiplicity (i.e., multiple dose comparisons and multiple endpoints) and to ensure that the alpha level is maintained at 1% will be made.

 Both TCZ treatment groups will be compared to the 52-week prednisone-taper group for all the study endpoints in an exploratory manner using the methodology as specified previously. Further exploratory analysis considering variables such as temporal artery biopsy status, compliance with 1990 ACR versus modified diagnostic criteria, type of disease flare (i.e., “cranial”, “PMR”, etc.), and concomitant MTX use will be also performed.

#### 2.13.2. Safety Analysis

The safety analyses will include all randomized patients who received at least one dose of study drug. Patients will be reported according to the treatment received. 

### 2.14. Sample Size and Power

A sample size of 100 patients in the A (162qw TCZ) group and 50 patients in both the B (162q2w TCZ) and C (placebo) groups will ensure at least 90% power to detect a difference in the proportion of patients in sustained remission at week 52 for both TCZ arms versus placebo at an alpha level of 0.01 (2 sided). This assumes that the absolute difference in the proportion of patients who are in sustained remission at 52 weeks is equal to 40% (assuming *ρ*_6-TCZ_ = 70% versus *ρ*_6-mCS_ = 30%). In addition, 50 patients will be included in the 52-week prednisone-tapering group (D).

Although a difference might still be identified, it should be noted that our study will not be powered to find a statistically significant difference between the TCZ arms and the 52-week prednisone-tapering arm (group D). However, we anticipate that data from patients in this fourth arm will provide valuable information regarding the optimal dosing regimens of CS in GCA and help evaluate the risk-benefit ratio of TCZ as a CS-sparing agent.

### 2.15. Data and Safety Monitoring

An independent Data and Safety Monitoring Board (DSMB) plans to review accumulated data on safety and efficacy at least twice a year and/or in an event-driven fashion as necessary.

### 2.16. Mechanistic Substudy

Peripheral blood will be collected at different timepoints to evaluate the impact of anti-IL-6R therapy on the concentrations of selected cytokines and chemokines. Genome-wide mRNA expression profiling will be done at baseline and 12 months. 

## 3. Discussion

The current standard of care for GCA, CS therapy, is a suboptimal treatment strategy. A high percentage of patients relapse upon weaning treatment and more than 85% of the patients suffer from CS-related side effects [7, 12, 14, 15, 44]. Persistently active vascular inflammation despite ongoing CS therapy has been demonstrated in animal models [32, 50] and confirmed in patients who lack overt clinical symptoms [51–53]. In addition, up to a quarter of all cases experience complications from large vessel inflammation (i.e., aneurysm) despite treatment with CS [53–56]. Thus, an unmet need exists in GCA for better and less toxic treatment alternatives. GiACTA will explore the hypothesis that TCZ is effective in maintaining GCA remissions and sparing CS. 

Controlled studies in GCA must address several challenges. First, although management guidelines have been created for large-vessel vasculitis and GCA [57, 58], the absence of standardization for CS therapy creates difficulties when comparing a new treatment approach. Second, the high morbidity burden related to GCA argues in favor of designing trials with endpoints that reflect clinically meaningful outcomes and justify the use of newer, sometimes expensive therapies. Third, since the development of the ACR classification criteria for GCA [59], rheumatology has incorporated new imaging modalities that often complement the diagnostic process of GCA (US, CTA, MRI/MRA, and PET-CT) [60]. For this reason, some patients currently diagnosed in clinical practice may not be captured by the 1990 criteria [61]. Moreover, the mechanism of action of TCZ creates specific problems that need to be considered *a priori* in order to prevent the unblinding of treatments that some expected laboratory changes could generate. Finally, although requiring a masked prednisone-taper phase that begins once patients reach 20 mg/day creates certain logistical challenges, this necessity has led to a novel trial design that will accurately assess the potential of TCZ for exerting CS-sparing effects in this disease. We address these issues, one by one. 

### 3.1. Defining the Standard of Care for Corticosteroid Use

Large vessel vasculitis (LVV) guidelines address the initial CS dose but do not standardize the rate of tapering in GCA [57]. The approach to treatment varies significantly among physicians. As a general rule in usual clinical practice, a newly diagnosed patient initially receives the equivalent of approximately 1 mg/kg of prednisone daily (*≈*60 mg/day). This dose is given until the reversible manifestations of the disease abate and the inflammatory markers normalize (*≈*4 weeks). The dose is then gradually tapered to reach a maintenance of 10 mg/day by 2.5–6 months, and 5 to 0 mg/day by 12–24 months (and sometimes longer) [5, 8]. Subjects already carrying the diagnosis of GCA whose symptoms recur upon CS tapering (relapsing/refractory GCA) typically receive a dose of 10 mg above the last dose that was able to control the disease, unless the severity of the flare warrants higher doses (e.g., threatened vision loss). Following control of the activity, a tapering regimen of variable length is instituted. The median cumulative CS dose of an American cohort of GCA patients followed for 10 years was 7.4 grams (mean daily dose 13.6 mg/day). This treatment led to significant CS-induced toxicity, yet was associated with a relapse rate of 48% [7]. More recently, a relapse rate of 40.8% and mean cumulative CS doses of 12.5 grams were demonstrated in a population-based study from Northwestern Spain [62].

In contrast to the variability of clinical practice that often leads to CS use extending over years, clinical trials have frequently used tapers over a period of six months. Unfortunately, the investigational agents employed to date in these studies have not been successful in reducing the number of disease flares in a meaningful manner, and annual relapse rates in some trials have been as high as 80% [14, 15]. 

To address the problem of variability in the standard of care, GiACTA will use two prednisone-tapering regimens as control arms. The six-month taper was created for the efficacy analysis, to test the ability of TCZ to maintain disease remission and spare CS. Since many patients in the course of usual care receive courses that are longer than six months, we will also enroll patients into a comparative 12-month prednisone-tapering arm (group D). It should be noted that the study will not be powered to find a statistically significant difference between the TCZ arms and the 12-month prednisone-tapering arm, but this group will provide substantial information on the natural history of CS-treated disease and serve for exploratory analysis. To prevent unmasking due to the different tapering protocols, both the patients and investigators will be blinded to the dose of prednisone given after the daily dose has been reduced to 20 mg/day (week 7) through the end of Part 1 (week 52). 

### 3.2. Identification of Clinically Meaningful Endpoints

The ideal agent for a chronic immune-mediated disease would restore tolerance and induce drug-free remissions following treatment. More realistically, a useful agent for GCA should be able to maintain disease remission, while decreasing the toxicity associated with CS therapy. CS-free sustained remission at 52 weeks, cumulative CS dose, CS-related AEs, CS-TCZ-free sustained remission, and quality of life measures comprise a set of meaningful endpoints that will rigorously test the efficacy of TCZ in GCA. Furthermore, assuming an absolute treatment effect difference of 40% between the experimental and the control groups, and setting the alpha level at 0.01 for the analysis of the primary endpoint will assure robustness of the results if the trial meets its primary outcome. 

### 3.3. GCA Diagnostic Criteria

We anticipate that the vast majority of subjects enrolled in GiACTA will meet the ACR 1990 classification criteria for the diagnosis of GCA [59]. However, as progress has been made in understanding the pathology of the disease and new vascular imaging modalities have emerged since 1990, we have created revised diagnostic criteria that are consistent with current clinical practice and consider the presence of evidence of large vessel vasculitis in cross-sectional imaging (MRA, CTA, PET-CT, or angiography) an important diagnostic feature. Because these radiologic tests have not yet been validated for the diagnosis of GCA, the results using this approach will be interpreted with caution, and we will perform a sensitivity analysis to evaluate the study outcomes in patients meeting the ACR classification criteria versus the overall study population. 

### 3.4. Prevention of Unblinding

To prevent unblinding due to expected rapid normalization of inflammatory markers or the occurrence of specific laboratory abnormalities sometimes observed during TCZ therapy (e.g., neutropenia, transaminase elevation), a safety assessor independent of the investigator/efficacy assessor will monitor the results of the following laboratory parameters: ESR, CRP, total white blood cell count (WBC), absolute neutrophil count (ANC), platelet counts, and aspartate aminotransferase (AST) and alanine aminotransferase (ALT) concentrations. The safety assessor will determine the indication for dose modification of TCZ (or TCZ placebo) following guidelines and risk mitigation strategies used for TCZ in the RA studies. 

We have considered the possibility that blinding the investigators to the levels of acute phase reactants could create problems during the longitudinal follow-up, since many clinicians rely at least in part on levels of these markers as guides to the degree of ongoing inflammation. As an example, the ESR and CRP values may help in differentiating non-specific musculoskeletal pain from a flare of PMR during CS dose reduction. However, the levels of acute phase reactants can be misleading, with both false-negative and false-positive results. Overtreatment with CS following excessive emphasis on the values of inflammatory markers places patients at risk for additional CS-related morbidity. For this reason, once patients are enrolled, only clearly defined signs and symptoms will be used as parameters of disease activity, and only investigators with expertise in the care of patients with GCA will participate.

### 3.5. Prednisone Blinding

To our knowledge, this is the first clinical trial in any disease to incorporate blinded CS regimens into its design. This strategy, which will permit an unbiased evaluation of the CS-sparing effects of TCZ, may be extrapolated to trials in other immune-mediated conditions, thereby helping to address an important need in the area of inflammation. 

### 3.6. Exploratory Analyses

The largest trial performed to date in GCA offers important opportunities for exploratory substudies. In this regard, analyzing the effect that IL-6 signaling blockade has on different components of the Th1, Th17 and Treg pathways may elucidate the mechanisms of action of TCZ and increase the understanding of the immunopathogenesis of this disease. Moreover, genome-wide transcriptome analysis may identify important pathogenic factors, new biomarkers, and gene signatures associated with critical clinical outcomes (e.g., relapse risk, response to therapy, and others).

## 4. Conclusion

In summary, the GiACTA study will assess the safety and efficacy of TCZ for the treatment of GCA. The use of CS dose blinding is unique in the field of clinical trials of immune-mediated disease and may be extended to other investigations in which the identification of a CS-sparing agent is important. Successful achievement of the primary outcome in this trial would mark the first demonstration of an effective alternative to continuous CS use as a strategy for maintaining remission in GCA.

## Figures and Tables

**Table 1:** Critical trial definitions.

Term	Definition
Revised GCA diagnosis criteria	(1) Age ≥50 years (2) History of ESR ≥50 mm/hour (3) And at least one of the following: (a) Unequivocal cranial symptoms of GCA (new onset localized headache, scalp or temporal artery tenderness, ischemia-related vision loss, or otherwise unexplained mouth or jaw pain upon mastication) (b) Unequivocal symptoms of polymyalgia rheumatica (PMR), defined as shoulder and/or hip girdle pain associated with inflammatory stiffness (4) And at least one of the following: (a) Temporal artery biopsy revealing features of GCA (b) Evidence of large-vessel vasculitis by angiography or cross-sectional imaging study such as magnetic resonance angiography (MRA), computed tomography angiography (CTA), or positron emission tomography-computed tomography (PET-CT)

New onset GCA	Diagnosis of GCA made within 6 weeks of baseline visit

Relapsing/refractory GCA	Diagnosis of GCA >6 weeks of baseline visit and active disease within 6 weeks of baseline visit

Active GCA	(1) At least one of the following within 6 weeks of baseline visit (a) Unequivocal cranial symptoms of GCA (new onset localized headache, scalp or temporal artery tenderness, ischemia-related vision loss, or otherwise unexplained mouth or jaw pain upon mastication) (b) Unequivocal symptoms of polymyalgia rheumatica (PMR), defined as shoulder and/or hip girdle pain associated with inflammatory stiffness (c) Other features judged by the clinician investigator to be consistent with GCA or PMR flares (i.e., new or worsened extremity claudication, fever of unknown origin) (2) And ESR ≥30 mm/hr or CRP ≥1 mg/dL

Remission	Absence of all symptoms attributable to active GCA and normalization of ESR (<30 mm/hr) and CRP (<1 mg/dL)

Flare	Recurrence of symptoms attributable to active GCA, with or without elevation of ESR and/or CRP

Sustained remission (SR)	(1) Absence of flare following remission by week 12 after randomization (2) And completion of the assigned prednisone taper (3) And not having required escape therapy at any time by week 52.

**Table 2:** Eligibility criteria.

Inclusion criteria	Exclusion criteria
(1) Diagnosis of GCA (2) Active disease within 6 weeks of baseline visit (3) Willing to receive antiplatelet therapy (4) Willing to receive treatment for prevention of glucocorticoid-induced osteopenia/osteoporosis	(1) Recent or incoming major surgery (2) Organ transplantation recipient (except corneas within 3 months prior to baseline visit) (3) Prior treatment with any of the following: (i) Investigational agents within 12 weeks of screening visit (ii) Cell depleting agents (i.e., anti-CD20) (iii) Alkylating agents including CYC (iv) Tocilizumab (v) HCQ, CsA, AZA, or MMF within 4 weeks of baseline (vi) Tumor necrosis factor inhibitors within 2–8 weeks of baseline depending on the agent (vii) Anakinra within 1 week of baseline (viii) MTX started within 6 weeks of study enrollment (ix) CS for other conditions other than GCA (4) History of severe allergic reactions to monoclonal antibodies (5) Evidence of serious uncontrolled concomitant disease (i.e., cardiovascular, respiratory, renal, endocrine, etc.) (6) Current liver disease that could interfere with the trial as determined by the physician investigator (7) History of diverticulitis, inflammatory bowel disease, or other symptomatic GI tract condition that might predispose to bowel perforation (8) Infections: (i) Active current or history of recurrent bacterial, viral, fungal, mycobacterial, or other infection (ii) Prior episode of major infection (iii) Active TB requiring treatment within the previous 3 years (iv) Untreated latent TB infection (LTBI) (9) Primary or secondary immunodeficiency (10) Malignancy (except basal and squamous cell carcinoma of the skin or carcinoma in situ of the cervix uteri that has been excised and cured) (11) Laboratory abnormalities: AST or ALT >1.5 × upper limit of normal (ULN), total bilirubin > ULN, platelet count <100 × 10^9^/L, hemoglobin <8.5 gr/dL, WBC count <3 × 10^9^/L, ANC <2 × 10^9^/L, ALC <0.5 × 10^9^/L, positive HBs antigen or positive HCV antibody

GCA: giant cell arteritis; CYC: cyclophosphamide; HCQ: hydroxychloroquine; CsA: cyclosporine A; AZA: azathioprine; MMF: mycophenolate mofetil; MTX: methotrexate; CS: corticosteroids; TB: tuberculosis; AST: aspartate aminotransferase; ALT: alanine aminotransferase; ULN: upper limit of normal; WBC: white blood cell; ANC: absolute neutrophil count; ALC: absolute lymphocyte count; HBs antigen: hepatitis B virus superficial antigen; HCV: hepatitis C virus.

**Table 3:** Standardized prednisone-taper protocols during GiACTA.

Weeks	Daily prednisone dose (mg) 26-week taper	Daily prednisone dose (mg) 52-week taper
1	60	60
2	50	50
3	40	40
4	35	35
5	30	30
6	25	25
7	20	20

After week 7, the CS dosing will be double-blinded		

8	15	17.5
9	12.5	17.5
10	12.5	15
11	10	15
12	9	12.5
13	8	10
14	7	10
15	6	10
16	6	10
17	5	9
18	5	9
19	4	9
20	4	9
21	3	8
22	3	8
23	2	8
24	2	8
25	1	7
26	1	7
27	CS placebo	7
28	CS placebo	7
29	CS placebo	6
30	CS placebo	6
31	CS placebo	6
32	CS placebo	6
33	CS placebo	5
34	CS placebo	5
35	CS placebo	5
36	CS placebo	5
37	CS placebo	4
38	CS placebo	4
39	CS placebo	4
40	CS placebo	4
41	CS placebo	3
42	CS placebo	3
43	CS placebo	3
44	CS placebo	3
45	CS placebo	2
46	CS placebo	2
47	CS placebo	2
48	CS placebo	2
49	CS placebo	1
50	CS placebo	1
51	CS placebo	1
52	CS placebo	1
